# Endophytic *Trichoderma* spp. can protect strawberry and privet plants from infection by the fungus *Armillaria mellea*

**DOI:** 10.1371/journal.pone.0271622

**Published:** 2022-08-01

**Authors:** Helen J. Rees, Jassy Drakulic, Matthew G. Cromey, Andy M. Bailey, Gary D. Foster

**Affiliations:** 1 School of Biological Sciences, University of Bristol, Bristol, United Kingdom; 2 Department of Plant Health, Royal Horticultural Society, Wisley, Surrey, United Kingdom; Banaras Hindu University, INDIA

## Abstract

*Armillaria mellea* is an important fungal pathogen worldwide, affecting a large number of hosts in the horticulture and forestry industries. Controlling *A*. *mellea* infection is expensive, labour intensive and time-consuming, so a new, environmentally friendly management solution is required. To this effect, endophytic *Trichoderma* species were studied as a potential protective agent for *Armillaria* root rot (ARR) in strawberry and privet plants. A collection of forty endophytic *Trichoderma* isolates were inoculated into strawberry (*Fragaria × ananassa*) plants and plant growth was monitored for two months, during which time *Trichoderma* treatment had no apparent effect. *Trichoderma*-colonised strawberry plants were then inoculated with *A*. *mellea* and after three months plants were assessed for *A*. *mellea* infection. There was considerable variation in ARR disease levels between plants inoculated with different *Trichoderma* spp. isolates, but seven isolates reduced ARR below the level of positive controls. These isolates were further tested for protective potential in *Trichoderma*-colonized privet (*Ligustrum vulgare*) plants where five *Trichoderma* spp. isolates, including two highly effective *Trichoderma atrobrunneum* isolates, were able to significantly reduce levels of disease. This study highlights the potential of plants pre-colonised with *T*. *atrobrunneum* for effective protection against *A*. *mellea* in two hosts from different plant families.

## Introduction

*Armillaria mellea* (Vahl) P. Kumm (Agaricales, Physalacriaceae), commonly known as Honey Fungus, is a destructive fungal plant pathogen found worldwide causing problems in forestry, horticulture, and agriculture [1]. It has a host range of over 500 species [2], although often regarded as a pathogen of trees, it can also infect shrubs and herbaceous plants, with new hosts recently recognised [3, 4]. Through a root-like network of foraging rhizomorphs, *A*. *mellea* can spread up to one metre a year [5] and can attack multiple hosts on one site in garden settings [6]. Symptoms of *Armillaria* infection include typical root rot symptoms and the characteristic mycelial fan formed below the bark at the stem base, with honey-coloured fruiting bodies sometimes visible in cases of severe infection [1, 7]. Chemical control of *Armillaria* root rot (ARR) is either ineffective [8, 9] or has been banned due to negative environmental impacts [1, 10] leaving only expensive and labour-intensive options for controlling the disease. A new and environmentally friendly option is desperately required to control ARR.

*Trichoderma* (Hypocreales, Hypocreaceae) species are ubiquitous soil fungi and can grow saprophytically on a range of carbon and nitrogen substrates [11]. Furthermore, *Trichoderma* spp. can form endophytic plant root-associations and are widely regarded as plant growth promoting fungi [12]. There are numerous and complex means by which *Trichoderma* can promote plant growth, for example, *T*. *harzianum* can access insoluble elements in the soil, thereby increasing nutrient availability to the plant [13]. Fungal volatile organic compounds (VOCs) can enhance growth promotion as demonstrated in lettuce (*Lactuca sativa*) by *T*. *asperellum* [14]. Upregulation of indole-3-aceteic acid (IAA) genes by *T*. *asperellum* in tea (*Camellia sinensis*) [15] suggest that growth promotion is a result of the production of growth inducing hormones. *Trichoderma* spp. have also been merited with the ability to increase photosynthetic potential in plants [16] which in turn results in increased plant growth.

*Trichoderma* spp. are of particular interest in this study for their potential as biological control agents. *Trichoderma* species have often been cited as a successful biological control agents against fungal plant pathogens, including fungi such as *Colletotrichum gloeosporioides*, *Calonectria pauciramosa*, *Fusarium oxysporum* f. sp. *radicis-cucumerinum*, *Rhizoctonia solani* and *Sclerotinia trifoliorum* [15, 17–20]. Like growth promotion, there are many ways by which *Trichoderma* species protect host plants from disease including up-regulation of disease resistance genes, induced systemic resistance, production of VOC’s, mycoparasitism and direct competition [12, 14, 15]. However, there have been reports of *Trichoderma viride* acting pathogenically on cucumber, pepper and tomato seeds and causing faster disease progression of *Fusarium circinatum* in *Pinus radiata* [21], therefore testing putative biocontrol agents is vital to ensure disease will not be enhanced. *Trichoderma* spp. have been tested for biological control of *Armillaria* spp. with some signs of success. *Trichoderma atrobrunneum*, *T*. *atroviride*, *T*. *hamatum*, *T*. *harzianum* and *T*. *virens* have been found to offer protection from ARR in strawberry (*Fragaria × ananassa*) plants, Turkey oak (*Quercus cerris*) and apple (*Malus domestica*) seedings when applied either directly to the roots [22] or via a *Trichoderma* sp. inoculated substrate [23–25]. In addition *Trichoderma* spp. have been trialled as a preventative treatment in eucalyptus (*Eucalyptus diversicolor*) stumps where inoculation with a *Trichoderma* spp. spore suspension could reduce the colonization of eucalyptus stumps by *A*. *luteobubalina* [26].

While previous studies have investigated the role of *Trichoderma* spp. to offer plant protection against ARR, this study focuses on using novel, endophytic *Trichoderma* spp. isolates. These endophytic *Trichoderma* spp. isolates were collected from the roots of heathy plants, considered susceptible to ARR, that were growing in close proximity to plants which had succumbed to *Armillaria* infection [27]. It was hypothesized that the root microbiome helped these susceptible plants evade infection.

This study aimed to identify native, endophytic *Trichoderma* spp. isolates with the potential to offer plant protection against ARR in pot-based experiments. The hypothesis was that inoculating plants with endophytic *Trichoderma* spp. would encourage healthy growth and reduce *Armillaria mellea* infection. In strawberry plants, reports have been made of root colonization by *Trichoderma* spp. for up to one year after inoculation [28]. In this study, strawberry plants were used as a host system to rapidly screen *Trichoderma* spp. isolates. Further investigations were performed in privet plants, which are highly susceptible to *A*. *mellea* infection [4, 6], to establish whether *Trichoderma* spp. could reduce ARR in two different susceptible plant families with either woody or herbaceous growth habits.

## Materials and methods

### Fungal strains

A collection of 40 endophytic *Trichoderma* spp. isolates were obtained from RHS Garden Wisley (Surrey, UK), as described by Rees *et al*. [27] and detailed S1 Table. Identification was confirmed through sequencing of the ITS1, *tef1* or *rpb2* gene regions to give reliable *Trichoderma* species ID [27]. Isolates were maintained on malt extract agar (MEA), sealed with parafilm and incubated at 20°C with a 8:16 h light: dark cycle. The *Armillaria mellea* isolate CG440, collected from privet in 2006 [6, 29, 30], was maintained on MEA at 25°C in the dark and was recently passed through strawberry plants to prevent a loss of pathogenicity due to repeated subculturing. Isolations from plant material were made onto malt rose Bengal agar (MRB) [27] for *Trichoderma* spp. and JJG agar [6] for *A*. *mellea*.

### Plant material

All strawberry (*Fragaria × ananassa* ‘Elsanta’ (F)) plants were sourced from R W Walpole Ltd (Kings Lynn, UK) as bare-rooted plants with the variety Elsanta chosen based on its susceptibility to fungal diseases. Strawberry plants were grown at the University of Bristol greenhouses at Old Park Hill (51.456310, -2.599110) with a consistent temperature of 15°C and 16 h day length. Colonization studies were carried out at the University of Bristol, Life Sciences Building GroDome facility (51.459201, -2.601155). All privet (*Ligustrum vulgare*) plants were propagated from semi-woody cuttings collected from a mature privet hedge located in Wisley Village, UK (51.322620, -0.474560) and propagated in a Hydropod (Greenhouse Sensation, UK) as per manufacturer’s instructions for four to six weeks. All privet plants and cuttings were maintained at 18°C day and 15°C night with 16 h day length and trials with *A*. *mellea* were conducted at the RHS Wisley Field Research Facility (51.322526, -0.474136). Plants were potted in 10 cm pots using a Levingtons compost: Silver Sand (3: 1) mix.

### *Trichoderma* spp. inoculation of plants

The method of *Trichoderma* spp. inoculation of plants was consistent for strawberry and privet plants. Spore suspensions of individual *Trichoderma* spp. isolates were prepared from 10-day old cultures, flooded with 2 ml of 5% Tween 20 solution and gently scraped with a sterile loop to release spores which were collected and stored at 4°C for a maximum of two days. *Trichoderma* spp. spore suspensions were prepared at a final concentration of 10^5^ conidia ml ^-1^. Plants were selected for uniformity and roots of each plant were dipped into 50 ml of *Trichoderma* spp. spore suspension for two minutes. Isolations were made from the growing medium (Levingtons compost: Silver Sand, 3: 1) to ensure there was no background *Trichoderma* colonization present. The growing medium was then mixed with the remaining spore suspension to ensure *Trichoderma* spp. colonization occurred and plants were planted in 10 cm pots (ca. 0.3 L growing medium per plant). Nil-*Trichoderma* plants were treated in the same manner, instead using a root dip of sterile distilled water (SDW).

### *Armillaria mellea* inoculation of plants

*Armillaria mellea* colonized hazel billets for use as inoculum for strawberry plants were prepared based on the method described by Desray *et al*. [31]. Hazel (*Corylus avellana*) stems measuring 13 mm—17 mm dimeter were cut into 50 mm billets, arranged vertically in 500 ml wide mouth jars (VWR), autoclaved three times (120°C for 30 mins) and submerged in carrot agar [3]. Four agar plugs of *A*. *mellea* CG440 were used to inoculate each container which was stored in a dark incubator at 20°C for three—six months. For inoculation of privet plants, billets were instead arranged horizontally in a single layer in a 0.65 L rectangular plastic container (Wilko, UK), autoclaved as before and submerged in 1% MEA. Six *A*. *mellea* CG440 plugs were used for inoculation of billets, which required one-month incubation in the dark at 20°C to achieve full colonization. All negative control billets were set up in the same way as the method described, but without inoculation of *A*. *mellea*. All containers were sealed and wrapped in clingfilm to avoid contamination. Prior to use, excess agar was scrapped off billets with a flame-sterilised scalpel. To inoculate plants with *Armillaria*, a 5 ml pipette tip was used to pierce the root-ball 50 mm from the plant root collar to create a space to insert the billet. Roots were permitted to be damaged to encourage *Armillaria* infection and highlight any protection offered by *Trichoderma* spp.. One *A*. *mellea* CG440 colonized hazel billet was inserted per plant and in *Armillaria*-free plants, an uncolonized hazel billet was inserted.

### Colonization of privet plants by *Trichoderma* spp.

To determine the efficiency and longevity of endophytic *Trichoderma* spp. colonization in privet roots, plants were inoculated with *Trichoderma* spp. and destructively sampled to isolate endophytic *Trichoderma* spp. across different time points. To ensure 1% Virkon solution was sufficient for surface sterilization during destructive sampling, an *in vitro* test was performed using *Trichoderma* spore suspensions prepared to a concentration of 4 x10^4^ conidia ml ^-1^. Five, 10-fold serial dilutions of *T*. *atrobrunneum* T17/11 conidia were gently mixed with an equal volume of 1% Virkon or SDW (control) for 2 mins before 20 μl of each suspension was spread onto MEA and incubated at 20°C in L: D (16: 8 h) conditions. The number of *T*. *atrobrunneum* T17/11 colonies were counted after two days. For each dilution series, the sterilizing efficiency of 1% Virkon was calculated as: 1 –(number of *Trichoderma* colonies grown after 1% Virkon treatment / number of Trichoderma colonies grown after water treatment) x 100. The sterilization was 83% effective at a 4 x10^3^ conidia ml ^-1^, 95% effective at 4 x 10^2^ conidia ml ^-1^ and 100% effective at a 4 conidia ml ^-1^.

To confirm the efficiency of *Trichoderma* spp. colonization in plant roots, isolations for *Trichoderma* spp. were made daily for one week. Privet plants were inoculated with *T*. *atrobrunneum* T17/11 and potted into silver sand. A sterile piece of filter paper lined the base of a 10 cm pot to prevent loss of sand and pots were placed on a saucer. Seven plants were treated, and a different plant was destructively sampled each day for seven days. Sand was washed from plant roots with tap water and randomly selected sections of root tips (5 mm) were surface sterilised in 1% Virkon for two mins then washed twice in SDW for two mins. Per plant, ten isolations were made and checked after one week for growth of *Trichoderma* spp.. To control for background *Trichoderma* colonization in uninoculated plants, isolations were made from a freshly rooted privet plant on the day of *Trichoderma*-inoculation.

The longevity of *Trichoderma* colonization in privet plants was tested over six weeks with three *Trichoderma* isolates: *T*. *hamatum* T17/10, *T*. *atrobrunneum* T17/11 and T17/15. Freshly rooted privet cuttings were inoculated with *Trichoderma* spp. isolates and grown in soil. The control group was mock inoculated with SDW. There were six replicates per treatment so that a different plant could be sampled each week. Over a six-week period one plant per treatment was destructively sampled weekly and isolations were made from the roots as described above. During week four, data could not be collected due to incubator malfunction.

### Screening of *Trichoderma* spp. isolates for growth promotion and protection against ARR in strawberry plants

*Trichoderma* spp. isolates were screened in strawberry plants to assess if the fungi conferred plant growth promoting properties. Treatments included two nil-*Trichoderma* controls: plants inoculated with or without *A*. *mellea*. Strawberry plants were arranged in a non-randomized block design with three replicates per treatment in early-February 2018 (126 plants in total). Measurements of leaf size (determined as length of leaf blade multiplied by the width at its widest point) were taken from a randomly selected mature leaf at 10-day intervals for 60 days at which point plant height was also measured.

Two months after inoculation with *Trichoderma* spp., strawberry plants were inoculated with *A*. *mellea*. Control treatments included two nil-*Trichoderma* treatments: a mock-inoculated *Armillaria*-free treatment and a treatment inoculated with only *Armillaria*. Plants were monitored fortnightly, and any fruits or flowers were removed. After three months plants were destructively harvested and assessed for disease using a disease severity index (DSI) (0–6 pt. scale). Aboveground symptoms of *Armillaria* infection included chlorosis and dieback. In strawberry roots, *Armillaria* infection resulted in lesions and mycelial fans. Disease severity was ascribed as follows: 0) Healthy plant. No above- or belowground symptoms of *A*. *mellea* infection and no re-isolation of *A*. *mellea* from plant material; 1) Aerial symptoms present. No belowground symptoms or re-isolation of *A*. *mellea*; 2) No above- or belowground symptoms of *A*. *mellea*. Presence confirmed by re-isolation of *A*. *mellea*; 3) No aerial symptoms present. Root lesions or *A*. *mellea* mycelial colonization visible in roots and confirmed by re-isolation; 4) Aerial symptoms present. Root lesions or *A*. *mellea* mycelial colonization visible in roots and confirmed by re-isolation; 5) Progressed aerial symptoms. Visible/heavy *A*. *mellea* mycelial colonization visible and lesions in roots and confirmed by re-isolation; 6) Dead plant with visible/heavy *A*. *mellea* mycelial colonization and lesions in roots and confirmed by re-isolation (S2 Table).

Inoculum billets were recovered and visually assessed for *A*. *mellea* colonization. Isolations were made for *A*. *mellea* and *Trichoderma* spp. onto selective media from each plant. *Trichoderma* spp. isolations were made primarily from root tissue which appeared healthy while *A*. *mellea* isolations were made from root and crown tissue with symptoms of infection such as presence of dark, water-soaked lesions or pale mycelial fans, where present.

### *Trichoderma* spp. for protection against ARR in privet plants

Privet plants were selected as a host system to investigate the protective potential of *Trichoderma* spp. against ARR due to the high susceptibility of privet to ARR infection. A selection of seven *Trichoderma* spp. isolates which resulted in reduced ARR in strawberry plants and one poor performing isolate from strawberry plants were inoculated into privet plants with nine replicates per treatment (*Trichoderma* spp. isolates: *T*. *virens* T17/02, *T*. *harzianum* isolates T17/03, T17/07 & T17/08, *T*. *hamatum* T17/10, *T*. *atrobrunneum* isolates T17/11 & T17/15, and *T olivascens* T17/42). The isolate *T*. *olivascens* T17/42, included as a poor performing isolate because all strawberry plants inoculated with T17/42 died. Plants were inoculated with *Trichoderma* spp. in mid-November 2018 and grown for one month prior to inoculation with *A*. *mellea* CG440. Two Nil-*Trichoderma* treatments were included as per previous experiments, these were *Armillaria*-only and *Armillaria*-free controls. Ninety privet plants were grown in total.

Privet plants were assessed for vitality once a fortnight. Any dead plants were harvested, and the roots dissected. If no *A*. *mellea* colonization was visible at this point, isolations for *Armillaria* were made. After nine months all surviving plants were destructively assessed for disease. Plant roots and stem bases were inspected for *A*. *mellea* colonization and scored according to a DSI on a 0–4 pt. scale. Aboveground symptoms of *Armillaria* infection included chlorosis and defoliation. In privet roots, *Armillaria* infection resulted in lesions and mycelial fans. Disease severity was ascribed as follows: 0) Healthy plant. No above- or belowground symptoms of *A*. *mellea* infection; 1) Aerial symptoms present, no below ground symptoms; 2) No aerial symptoms, root lesions or visible *A*. *mellea* mycelial colonization in roots; 3) Aerial symptoms present and root lesions or visible *A*. *mellea* mycelial colonization in roots; 4) Dead plant with visible/heavy *A*. *mellea* mycelial colonization or lesions in roots (S3 Table). Isolation for *A*. *mellea* was only made from non-symptomatic root tissue. Presence of rhizomorphs in the soil was recorded and billets were visually assessed for *A*. *mellea* persistence.

### Statistical analyses

All statistical analyses were carried out using R (v 4.1.0) with the additional packages ‘tidyverse’ [32] and ‘emmeans’ [33]. Graphs were created in ‘ggplot2’ [34] using the package ‘ggrepel’ [35].

Growth promotion by *Trichoderma* sp. in strawberry plants was assessed with a one-way repeated measures ANOVA with pairwise comparisons. The null hypothesis assumed no significant difference in leaf size or plant height between *Trichoderma* spp. treatments and controls. For the DSI of both privet and strawberry plants data was not normally distributed and could not be transformed to achieve normality thus a Kruskal-Wallis test was used to determine whether *Trichoderma* spp. isolates affected the DSI of strawberry plants. The null hypothesis assumed *Trichoderma* spp. would not affect ARR severity.

## Results

### *Trichoderma* spp. colonization of privet

*Trichoderma atrobrunneum* T17/11 colonization after inoculation was highly efficient with 100% *Trichoderma* re-isolation from each root segment over seven days. In contrast, in the control root sampled on ‘Day 0’, no *Trichoderma* was isolated.

Over six weeks, the average *Trichoderma* isolation efficiency was 92.2% for *T*. *hamatum* T17/10, 93.5% for *T*. *atrobrunneum* T17/11 and 98% for *T*. *atrobrunneum* T17/15. At six weeks post inoculation, the minimum efficiency of *Trichoderma* re-isolation was 80% (*T*. *hamatum* T17/10) (S1 Fig). Across the six-week sampling period just one *Trichoderma* colony was isolated from the *nil-Trichoderma* control (10% of the isolations during week three).

### Potential growth promotion and ARR protection by *Trichoderma* spp. in strawberry plants

Over the 60-day time course, leaf size increased from 2.69 ± 0.86 to 6.79 ± 1.18, where time had a significant effect on leaf size (one-way repeat measure ANOVA; F_5, 40_ = 3.0684, p < 0.001). Individual *Trichoderma* spp. treatments had no significant effect (pairwise comparisons; p > 0.9) compared to nil*-Trichoderma* controls. There was also no significant effect of *Trichoderma* treatments on strawberry plant height (one-way ANOVA; F_40, 80_ = 1.73, p > 0.05) compared to nil*-Trichoderma* controls. One plant each from *T*. *virens* T17/02 and *T*. *atrobrunneum* T17/11 treatments died prior to *Armillaria* inoculation and both were removed from the analysis.

### Potential protection from ARR in strawberry plants by *Trichoderma* spp.

After 60 days since inoculation with *A*. *mellea*, a total of 33 out of 117 strawberry plants pre-inoculated with *Trichoderma* spp. died (S1 Table). In sixteen *Trichoderma* spp. treatments, all strawberry plants survived to the end of the experiment. In a further 15 *Trichoderma* spp. treatments two plants (of three) survived. In six *Trichoderma* spp. treatments two plants died and in two *Trichoderma* spp. treatments all plants died (*T*. *hamatum* T17/33 & *T*. *olivascens* T17/42). Of the *Armillaria*-only control plants half of the strawberry plants died (three out of six). No *Armillaria*-free plants died in the experiments.

*Trichoderma* sporulation was prolific on the *Armillaria* hazel billet inoculated into two plants with *T*. *hamatum* T17/10 and one with *T*. *harzianum* T17/06. A small amount of *Trichoderma* sporulation was observed on billets from one plant inoculated with *T*. *virens* T17/02, *T*. *spirale* T17/24 and *T*. *atrobrunneum* T17/11, and in one plant treated with *T*. *atrobrunneum* T17/12 sporulation occurred in the growing medium.

**Fig 1:**
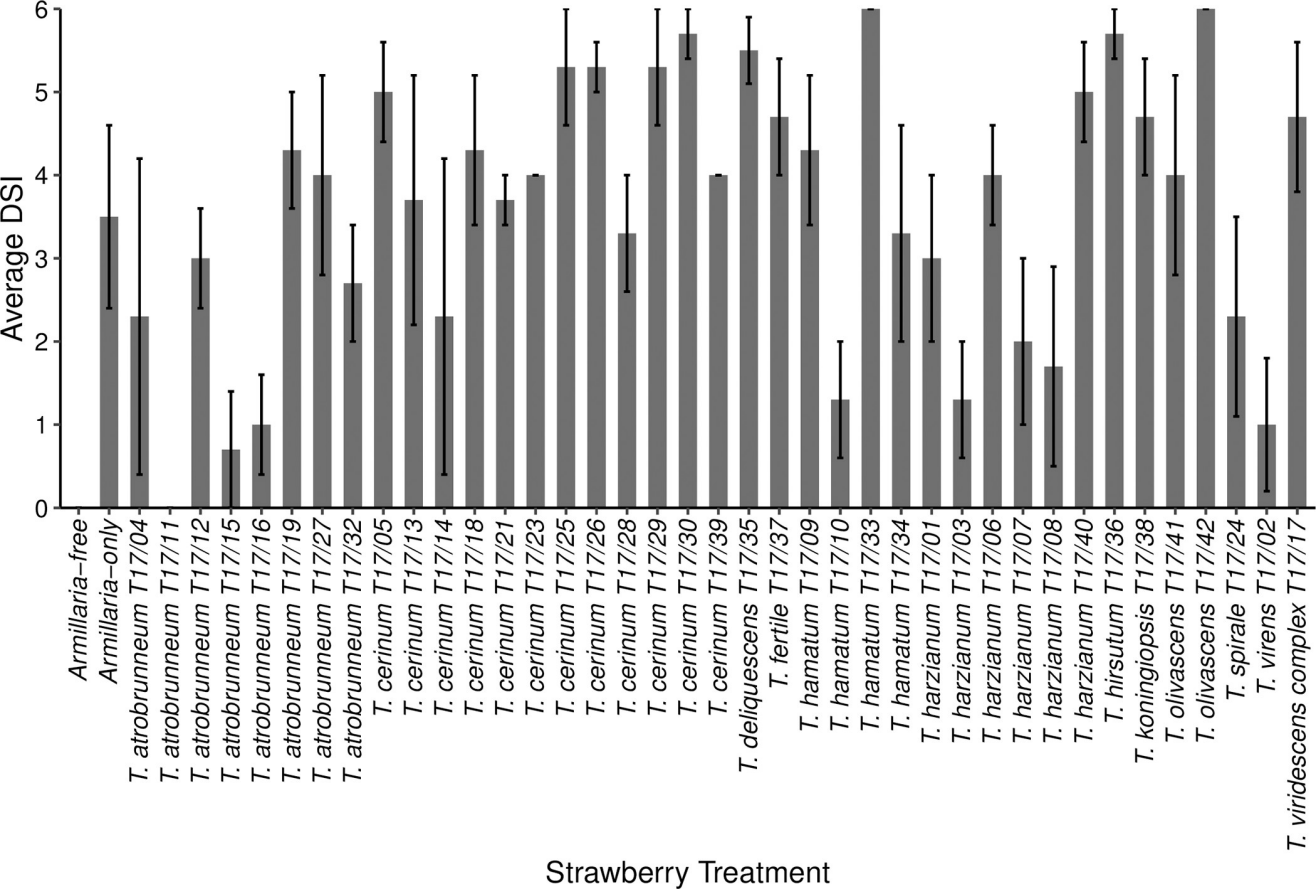
The average disease severity index (DSI) of *Armillaria mellea* CG440 infection in strawberry plants after three months in plants pre-colonised with *Trichoderma* spp. The DSI was measured on a scale from 0–6, where 0 = healthy plants. The error bars represent the standard error of the mean (n = 3).

Plants treated with *T*. *atrobrunneum* T17/11 had the lowest DSI (DSI = 0 ± 0; n = 2). The average DSI of *Armillaria*-only infected plants was 3.5 ± 1.1 (Fig 1) and no infection was recorded in *Armillaria*-free controls (DSI = 0 ± 0). The DSI for nil-*Trichoderma* treatments (those with or without *Armillaria*) did not significantly differ (Kruskal-Wallis: χ^2^ = 71.45, DF = 40, p > 0.05) and *Trichoderma* treatment did not significantly differ from the *Armillaria-*only or *Armillaria-*free controls.

Seven isolates whose DSI was lower (although not significantly lower) than that of the *Armillaria*-only control (DSI = 3.5 ± 1.1) were considered of interest for further study into plant protection against ARR. These isolates were: *T*. *virens* T17/02 (DSI 1.0 ± 0.8), *T*. *harzianum* T17/03 (DSI 1.3 ± 0.7), *T*. *harzianum* T17/07 (DSI 2.0 ± 1.0), *T*. *harzianum* T17/08 (DSI 1.7 ± 1.2), *T*. *hamatum* T17/10 (DSI 1.3 ± 0.7), *T*. *atrobrunneum* T17/11 (DSI 0.0 ± 0.0) and *T*. *atrobrunneum* T17/15 (DSI 0.7 ± 0.7). All plants for *T*. *olivascens* T17/42 (DSI 6.0 ± 0.0) died, so this treatment was included for further study as an example of a poorly performing isolate.

### Recovery of *Armillaria mellea* and *Trichoderma* spp. from strawberry plants

**Fig 2:**
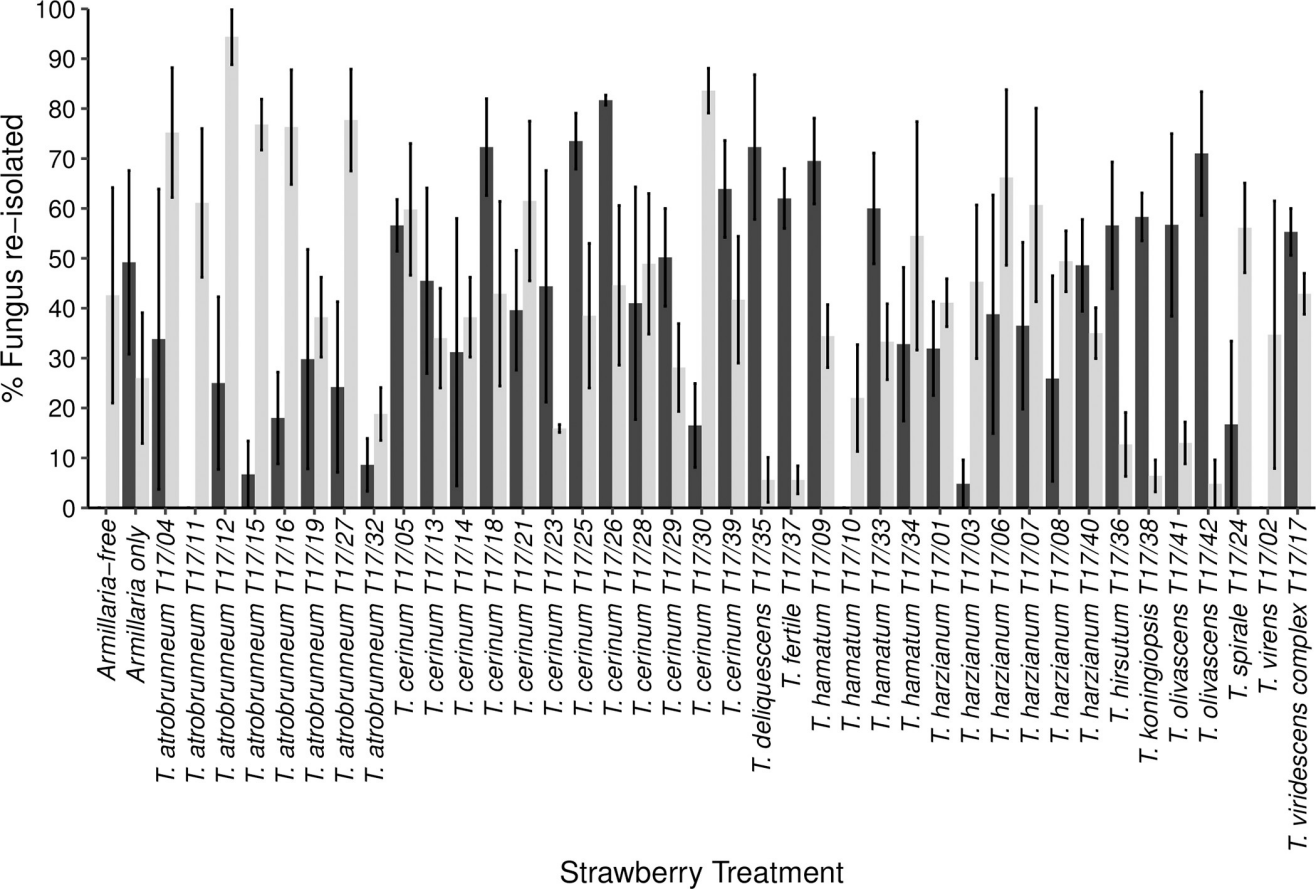
Re-isolation (percentage) of *Armillaria mellea* (black) and *Trichoderma* spp. (grey) from strawberry roots three months post inoculation with *A*. *mellea*.

Recovery of *A*. *mellea* from the *Armillaria* only control was 49.2%. Where plants were considered healthy, no *Armillaria* could be isolated including from the *Armillaria*-free control. From plants which had died from ARR (including *Armillaria*-only plants) successful re-isolation of *A*. *mellea* ranged from 38.4–98.3%. There was no significant difference in re-isolation of *Armillaria* between different treatments (Kruskal-Wallis: χ^2^ = 68.58, DF = 40, p > 0.05). *Armillaria* recovery was highest from plants inoculated with *T*. *cerinum* T17/26 (81.7% ± 0.9) and *T*. *cerinum* T17/25 (73.5% ± 5.6). No *Armillaria* was recovered from plants inoculated with *T*. *virens* T17/02, *T*. *hamatum* T17/10 and *T*. *atrobrunneum* T17/11 (0% ± 0) (Fig 2).

Attempts were made to isolate *Trichoderma* spp. from all plants, including nil*-Trichoderma* plants. Recovery of *Trichoderma* spp. from nil*-Trichoderma* plants was 42.6% in *Armillaria*-free and 26% in *Armillaria* only plants. There was no significant difference in re-isolation of *Trichoderma* spp. between different treatments (Kruskal-Wallis: χ^2^ = 74.15, DF = 40, p > 0.05). *Trichoderma* re-isolation was highest from plants inoculated with *T*. *atrobrunneum* T17/12 (94.4% ± 5.5) and *T*. *cerinum* T17/30 (83.6% ± 4.5) and lowest for *T*. *olivascens* T17/42 (4.8% ± 4.8) and *T*. *deliquescens* (T17/35; 5.6% ± 4.5) (Fig 2).

### Potential protection from ARR in privet plants by *Trichoderma* spp.

**Fig 3:**
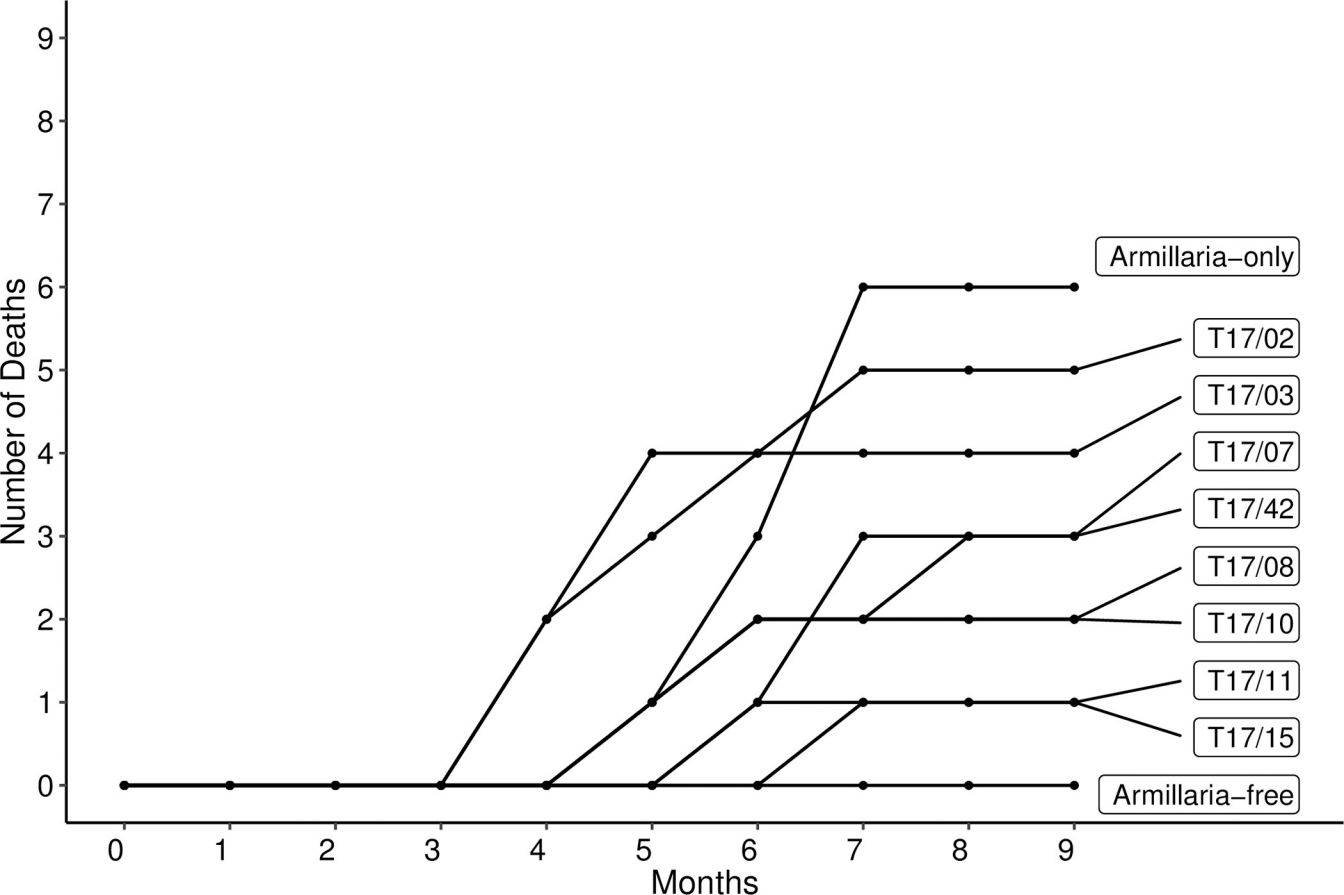
Number of privet plant deaths by *Armillaria mellea* CG440 over nine months. Treatments included two nil*-Trichoderma* controls (with and without *Armillaria*) and eight *Trichoderma* isolates (n = 9). *Trichoderma* species included *T*. *atrobrunneum* (T17/11 and T17/15), *T*. *hamatum* (T17/10), *T*. *harzianum* (T17/03, T17/07, T17/08), *T*. *olivascens* (T17/42) and *T*. *virens* (T17/02).

In the absence of *Trichoderma* spp. application, the first privet plants succumbed to *Armillaria mellea* infection by month five, and by seven months since inoculation six of the nine plants had died. Plant deaths occurred sooner in the presence of *T*. *virens* T17/02 or *T*. *harzianum* T17/03, first occurring at month four, and by month seven five of the nine plants had died for T17/02 and four for T17/03 (Fig 3). Thus, *Armillaria* infection started earlier, and these isolates showed little, if any, protective effect. In contrast, disease progression was delayed by the other *Trichoderma* spp. isolates, and mortality was reduced (Fig 3), suggesting that these may have a protective effect against ARR.

**Fig 4:**
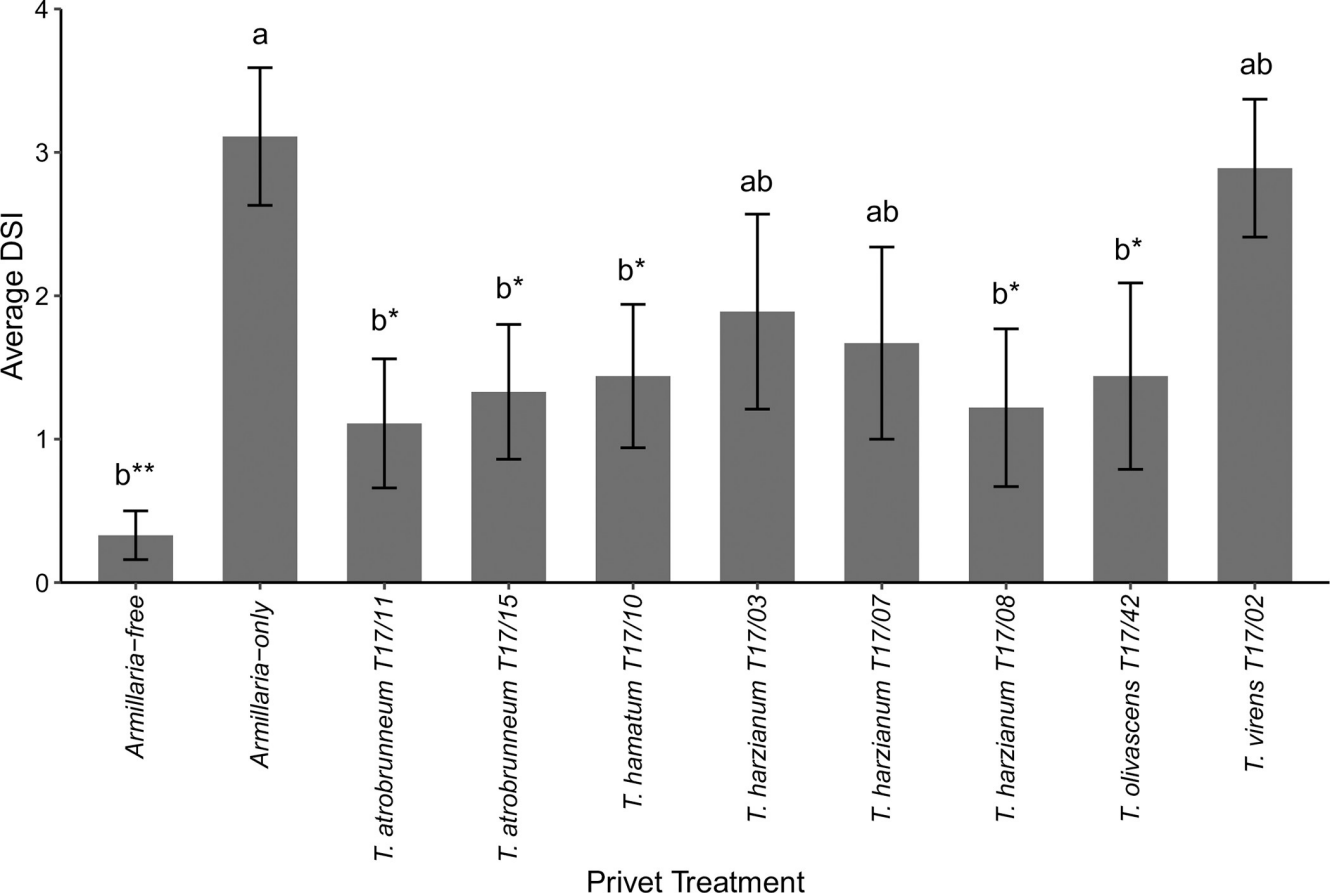
The average disease severity index (DSI) of *Armillaria mellea* CG440 infection in privet plants after nine months in plants pre-colonised with *Trichoderma* spp. The error bars represent the standard error of the mean (n = 9). a, b represent statistical groups (Kruskal-Wallis) and the level of significance is indicated as * p < 0.05, ** p < 0.01, *** p < 0.001.

Five *Trichoderma* spp. isolates (*T*. *harzianum* T17/08, *T*. *hamatum* T17/10, *T*. *atrobrunneum* T17/11 & T17/15 and *T*. *olivascens* T17/42) had a significantly lower DSI compared to the *Armillaria*-only control (Kruskal-Wallis with Bonferroni correction: χ^2^ = 17.86, DF = 9 p < 0.05) (Fig 4) showing evidence of ARR control by these *Trichoderma* spp.. The isolate *T*. *olivascens* T17/42, included as a ‘bad’ isolate because all strawberry plants inoculated with T17/42 died, had a significantly lower (p < 0.05) DSI than the *Armillaria*-only control in privet plants. Of *Armillaria-*infected plants, the treatments with the lowest mean DSI (0–4 pt. scale; n = 9) were *T*. *atrobrunneum* T17/11 (1.1 ± 0.45) and T17/15 (1.3 ± 0.45) (Fig 3). The highest DSI was recorded for *Armillaria*-only plants (3.1 ± 0.48) where six plants died. Privet plants inoculated with *T*. *virens* T17/02 had the highest DSI (2.8 ± 0.48) of *Trichoderma-*treated plants where five plants died, three had *A*. *mellea* mycelial fans in the roots but showed no above-ground symptoms, and one was healthy. No *Armillaria-*free plants died and their average DSI was 0.33 ± 0.17. Rhizomorphs were present in the soil of 90% of plants with *A*. *mellea* inoculation.

## Discussion

Results from this study suggest that endophytic *Trichoderma* spp., particularly isolates of *T*. *atrobrunneum* (F.B. Rocha, P. Chaverri & W. Jaklitsch), were able to protect plants exposed to a high inoculum pressure from *Armillaria* root rot (ARR) for several months. The protection was successful in two diverse hosts which are both highly susceptible to ARR [2–4, 6]: privet, a woody host, and strawberry, a herbaceous one. The best performing isolates in both hosts were *Trichoderma atrobrunneum* T17/11 and T17/15. Survival was 91% (23 plants, eight isolates) in strawberry plants (compared to 50% in control plants) and 89% (18 plants, two isolates) in privet plants (compared to 33% in control plants) inoculated with *T*. *atrobrunneum* prior to *Armillaria mellea* indicating that this species may have a role as an antagonist towards *A*. *mellea*.

Following root inoculation with T17/11 and T17/15, plus addition of the root-dip residues to the growing medium, no strawberry plants (out of 2 and 3 respectively) and just one privet plant (out of 9) died over three and nine months respectively after inoculation with *A*. *mellea*. The presence of *A*. *mellea* rhizomorphs in the growing medium of 90% of privet plants at harvest shows that the pathogen was active in the rootzone but was unable to penetrate the host roots and cause disease when these *Trichoderma* isolates were present. Successful disease evasion in both hosts shows that these *Trichoderma* have the potential to form beneficial associations with hosts as a generalist and may not be bound by restrictive host preferences. This conclusion is further supported when considering that they were obtained from the roots of *Viburnum bodnantense* and *Quercus* sp., respectively (S1 Table).

From the wide range of isolates screened in strawberry, inoculation from 14 other *Trichoderma* spp. isolates also prevented plant deaths from ARR after three months, but the visual index was able to discriminate between the most protective treatments. This supports the hypothesis that these isolates were contributing to disease evasion of the plants they were inoculated into and indicates that this trait can occur among several *Trichoderma* species. This is in line with reports that two isolates of *T*. *harzianum* s.s. controlled ARR in strawberry when applied to the growing medium [23].

In contrast to results from strawberry plants inoculated with isolates *T*. *virens* T17/02 and *T*. *harzianum* T17/03 in privet plants, these two isolates gave the poorest protection. Given these two isolates were obtained directly from soil rather than from within roots, it is possible they are unable to form endophytic associations in privet. Variable success of *T*. *harzianum* to protect against ARR is reported in the literature as well. The commercially available *T*. *harzianum* strain, ‘Trianum’, was found to have some potential to reduce *A*. *mellea* infection of strawberry by 13% - 67% based on visual assessment of above ground plant health [36]. Another study looked at protection offered to strawberry plants from ARR by *T*. *harzianum* s.s. strains pathogenic to commercial mushrooms. Between *T*. *harzianum* isolates the survival rate of plants varied from 25% to 83% [23] suggesting that like *in vitro* studies [27, 37, 38], antagonism and the ability to protect against ARR varies greatly between isolates, and is not simply a trait shared by species within the *T*. *harzianum* clade.

Rees *et al*. [27] studied the *in vitro* interaction between *A*. *mellea* and the eight *Trichoderma* spp. isolates selected for further study *in planta*. While *T*. *atrobrunneum* T17/11 and T17/15 performed well in plants, in culture, these isolates were unable to prevent outgrowth of the pathogen when re-isolated from *A*. *mellea* colonised hazel disks. Additionally, similar disease levels in privet were observed for *T*. *virens* T17/02 compared to infected controls without *Trichoderma*, but *in vitro* this isolate could inhibit growth of two *A*. *mellea* isolates colonized in hazel disks. Using *in vitro* assays, Rees *et al*. [27] speculate that these *Trichoderma* spp. could be degrading hyphae of *Armillaria*. The differences between *in vitro* antagonism and interactions in plant-based studies between the same *A*. *mellea* and *Trichoderma* spp. isolates highlights the importance of conducting laboratory and plant-based assays to develop a full understanding of protective potential.

In a garden setting, to control ARR it is advised to leave the ground fallow for six months to a year to allow fragments of rhizomorphs to die off following removal of infected roots and cultivation of the soil (Beal, unpublished data). Therefore, a long duration of protection will be of great benefit to gardeners who would prefer to replant into beds where gaps are formed due to cases of ARR. We propose that the endophytic colonization of host plants with *Trichoderma* spp. may form a long-term association and promote healthy growth to enable vulnerable plants, such as strawberry and privet, to evade *Armillaria* infection. Additionally, we hypothesise that young plants will be supported though the vulnerable establishment phase when they are most susceptible to ARR. To ensure this is achievable, a long-term study (minimum three years) should be conducted to better understand the colonization behaviour of *Trichoderma* spp. and whether long-term protection from *Armillaria* is possible. We hypothesise that endophytic association will promote longer duration of the association with the host plant and confer longer-lasting protection from ARR.

The endophytic nature of the association between host plants and the *Trichoderma* spp. isolates was demonstrated in this work. This study documents the first evidence for endophytic colonization by *Trichoderma* spp. in privet roots, whereas in strawberry an endophytic association with *Trichoderma* sp. has previously been shown [28]. Our method revealed that 1% Virkon was able to kill *Trichoderma* spp. that occurred on the outside of roots, thus re-isolations of *Trichoderma* spp. from sterilized root samples demonstrated that the fungi had dwelled within the roots as endophytes. The plants from which these *Trichoderma* isolates were originally collected from were neither privet nor strawberry, but instead the collection originated from a range of ornamental horticultural plants [27], and so indicate that they could form endophytic associations with a wide range of plants.

Endophytic colonization of grapevine rootstocks by *T*. *atroviride* has been reported within three days post inoculation [39] and in maize after five days colonization by *T*. *virens* [40]. In this study, *T*. *atrobrunneum* was found to colonize privet roots with 100% efficiency throughout the first seven days post inoculation. Pre-germinated conidia and mature hyphae were likely present in the spore suspension used in this study, since spore suspensions were unfiltered after collection from Petri dishes as described by Ruano-Rosa *et al*. [41] which, potentially, allowed faster colonization of roots. In addition, this study found that colonization of privet plants by *T*. *atrobrunneum* or *T*. *hamatum* lasted for at least six weeks, when sampling stopped.

In colonization assays with *Trichoderma* spp., some studies have reported *Trichoderma sp*. colonization in control plants that had not been inoculated with *Trichoderma* spp.. Cripps-Guazzone *et al*. [42] reported *T*. *atroviride* isolation at a low level (20%) from ryegrass without prior *Trichoderma* inoculation. Presence of *Trichoderma* sp. in nil-*Trichoderma* controls was also noted in the privet colonization assay in this study where *Trichoderma* sp. was isolated from 10% of the samples from one plant. After five months of *Trichoderma* colonization in strawberry plants, re-isolation of *Trichoderma* sp. from nil-*Trichoderma* plants reached 42.6%. The *Trichoderma* species isolated are thought to be a result of air-borne contaminants, background *Trichoderma* spp. in compost or water-splash from neighbouring plants kept in the greenhouse but indicates that this genera of fungi are well-suited to colonizing the roots of plants and can do so without causing negative consequences for plant health. There was no difference in growth between strawberry plants (measured by leaf size) with or without *Trichoderma* spp. inoculation. This result differs from expectations based on previous work that showed *Trichoderma* spp. can promote plant growth in a one-year period [28]. The use of bare-rooted plants in this study may explain this result, as such plants are cultivated to burst into vigorous growth upon planting, limiting the chance of detecting growth promotion by *Trichoderma* spp. in the initial growth stages of the plant. Thus, a longer timeframe may be needed to show any growth benefits of the *Trichoderma* spp.

During the initial screening trial in strawberry, some isolates appeared to stimulate ARR compared to controls without *Trichoderma* spp. amendment. Amaral *et al*. [21] reported enhanced disease of *Pinus radiata* seedlings inoculated with *T*. *viride* followed by *Fusarium circinatum*. Following investigation, they concluded that the time interval between *Trichoderma* and pathogen inoculation needed to be of a sufficient length or disease symptoms were enhanced instead of reduced. In this study, some strawberry plants appeared to have heightened disease severity an effect which occurred across a range of *Trichoderma* isolates and species. Possible mechanisms for this effect include suppression of plant immune responses while the *Trichoderma* spp. achieves endophytic colonization [21]. Gaining further insights into the effect on the plant during *Trichoderma* spp. colonization is required. It is likely that inoculation with *Trichoderma* spp. several months prior to planting is important. This practice could be carried out in nurseries on young plants or rooted cuttings to allow *Trichoderma* spp. colonization to stabilize before planting out. It is therefore necessary to gain a more detailed understanding of the colonization time required by *Trichoderma* spp. to begin acting as a protectant prior to introduction of *A*. *mellea*, and whether this time varies by host type or age.

The impact of adding new *Trichoderma* isolates into an environment could change the pre-existing microbial communities, and while we are deliberately altering the root microbiome of the plant, the fungi may be able to escape into the soil. On one hand, *Trichoderma* is a ubiquitous fungus found in soils, plant foliage and roots [12] thus introducing, a new isolate should not overtly affect the microbial community in the long-term. However, *Trichoderma* spp. have a range of mechanisms pathogenic towards other fungi which should be considered. The addition of *T*. *atroviride* to the soil in a vineyard found no long-lasting detrimental effects on the microbial communities in the soil [43]. Before the novel *Trichoderma* spp. isolates in this study are enrolled on a large scale to protect plants from ARR, investigations should consider how aggressive the isolates are towards the general microbial community to ensure no lasting damage is caused.

In conclusion, this work has demonstrated that *Trichoderma* spp., particularly *T*. *atrobrunneum*, could be used to protect young plants from death and damage by ARR, caused by the aggressive pathogen *A*. *mellea*, and that these fungi are capable of doing so over several months by forming an endophytic association within plant roots. Endophytic colonization of privet by *T*. *atrobrunneum* occurs rapidly and lasts for at least six weeks but we expect it to be a stable association for much longer as we reported in strawberry plants where colonization persists for at least five months. Further investigations should consider the effect of microbial communities on the potential of *Trichoderma* to carry out its protective potential, and the environmental implications of introducing a *Trichoderma* spp. into soils.

## Supporting information

S1 Fig Percentage (%) recovery of *Trichoderma* spp. from privet roots grown in soil over six weeks. Privet plants were inoculated with three *Trichoderma* treatments (*T*. *hamatum* T17/10, *T*. *atrobrunneum* T17/11 and *T*. *atrobrunneum* T17/15) and included a *Trichoderma-*free control. *Trichoderma* spp. isolations were made from the roots of one plant.
(PDF)

S1 Table Details of *Trichoderma* spp. ID, including plants each was originally isolated from, adapted from Rees *et al*. [22]. The number of strawberry plant deaths and average disease severity index from strawberry plants (n = 3) treated with *Trichoderma* spp. and inoculated with *Armillaria mellea* CG440 is presented with the standard error.
(PDF)

S2 Table Disease Severity Index (0–6 pt. scale) descriptions for *Armillaria mellea* infection of strawberry after three months. (PDF)

S3 Table Disease Severity Index (0–4 pt. scale) descriptions for *Armillaria mellea* infection of privet after nine months. Isolation for *Armillaria* was only made from non-symptomatic tissue, if *Armillaria* was cultured this was classified as detectable colonization.
(PDF)
