# Supplementary material for: Endophytic Trichoderma spp. can protect strawberry and privet plants from infection by the fungus Armillaria mellea
Source: PLoS One. 2022 Aug 1;17(8):e0271622. doi: 10.1371/journal.pone.0271622 (PMC9342734; doi:10.1371/journal.pone.0271622)
Supplement: S1 Fig — Privet plants were inoculated with three Trichoderma treatments (T. hamatum T17/10, T. atrobrunneum T17/11 and T. atrobrunneum T17/15) and included a Trichoderma-free control. Trichoderma spp. isolations were made from the roots of one plant. (PDF) [file pone.0271622.s001.pdf]

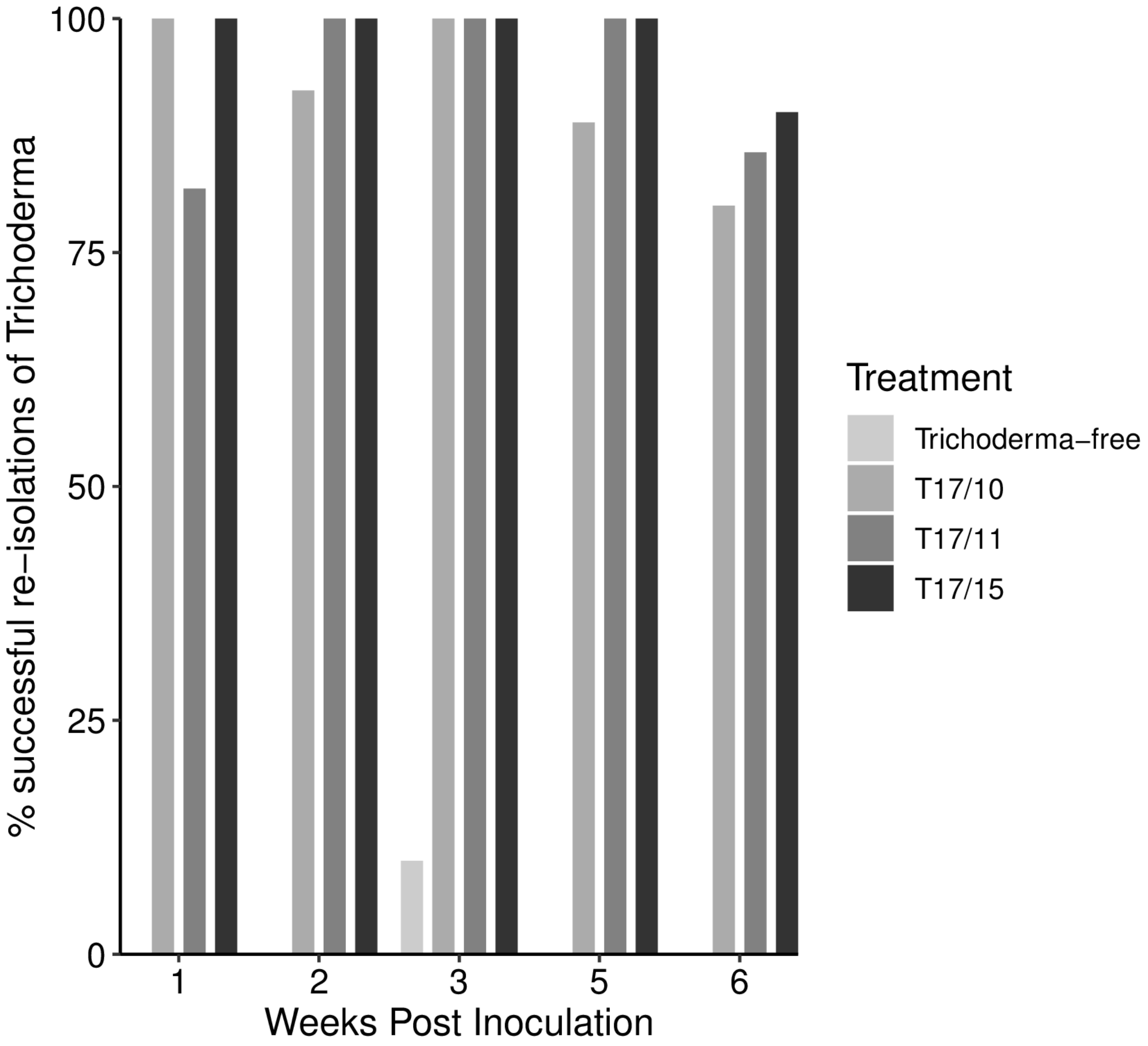

S1 Fig. Percentage (%) recovery of *Trichoderma* spp. from privet roots grown in soil over six weeks. Privet plants were inoculated with three *Trichoderma* treatments (*T. hamatum* T17/10, *T. atrobrunneum* T17/11 and *T. atrobrunneum* T17/15) and included a *Trichoderma*-free control. *Trichoderma* spp. isolations were made from the roots of one plant.
