# Supplementary material for: Endophytic Trichoderma spp. can protect strawberry and privet plants from infection by the fungus Armillaria mellea
Source: PLoS One. 2022 Aug 1;17(8):e0271622. doi: 10.1371/journal.pone.0271622 (PMC9342734; doi:10.1371/journal.pone.0271622)
Supplement: S1 Table — The number of strawberry plant deaths and average disease severity index from strawberry plants (n = 3) treated with Trichoderma spp. and inoculated with Armillaria mellea CG440 is presented with the standard error. (PDF) [file pone.0271622.s002.pdf]

S1 Table. Details of *Trichoderma* spp. ID, including plants each was originally isolated from, adapted from Rees *et al.* [22]. The number of strawberry plant deaths and average disease severity index from strawberry plants (n = 3) treated with *Trichoderma* spp. and inoculated with *Armillaria mellea* CG440 is presented with the standard error.

Isolate	*Trichoderma* ID	Isolate source	Number of strawberry plant deaths	DSI
T17/01	*T. harzianum*	*Viburnum carlesii*	0	3.0 ± 1.0
T17/02 [a]	*T. virens*	Soil/debris	0	1.0 ± 0.8
T17/03 [a]	*T. harzianum*	Soil/debris	0	1.3 ± 0.7
T17/04	*T. atrobrunneum*	*Viburnum carlesii*	1	2.3 ± 1.9
T17/05	*T. cerinum*	*Citrus trifoliata*	1	5.0 ± 0.6
T17/06	*T. harzianum*	*Quercus* sp.	1	4.0 ± 0.6
T17/07 [a]	*T. harzianum*	*Quercus* sp.	0	2.0 ± 1.0
T17/08 [a]	*T. harzianum*	*Quercus* sp.	0	1.7 ± 1.2
T17/09	*T. hamatum*	*Sorbus aria 'Lutescens'*	1	4.3 ± 0.9
T17/10 [a]	*T. hamatum*	*Sorbus aria 'Lutescens'*	0	1.3 ± 0.7
T17/11 [a]	*T. atrobrunneum*	*Quercus* sp.	0	0.0 ± 0.0
T17/12	*T. atrobrunneum*	*Quercus* sp.	0	3.0 ± 0.6
T17/13	*T. cerinum*	*Quercus* sp.	1	3.7 ± 1.5
T17/14	*T. cerinum*	Sorbus aria 'Lutescens'	1	2.3 ± 1.9
T17/15 [a]	*T. atrobrunneum*	*Viburnum bodnantense*	0	0.7 ± 0.7
T17/16	*T. atrobrunneum*	*Viburnum bodnantense*	0	1.0 ± 0.6
T17/17	*T. viridescens complex*	*Betula pendula*	1	4.7 ± 0.9
T17/18	*T. cerinum*	*Betula pendula*	1	4.3 ± 0.9
T17/19	*T. atrobrunneum*	*Viburnum bodnantense*	0	4.3 ± 0.7
T17/21	*T. cerinum*	*Betula pendula*	0	3.7 ± 0.3
T17/23	*T. cerinum*	*Betula pendula*	0	4.0 ± 0.0
T17/24	*T. spirale*	*Betula pendula*	0	2.3 ± 1.2
T17/25	*T. cerinum*	*Betula pendula*	2	5.3 ± 0.7
T17/26	*T. cerinum*	*Betula pendula*	2	5.3 ± 0.3
T17/27	*T. atrobrunneum*	*Viburnum bodnantense*	1	4.0 ± 1.2
T17/28	*T. cerinum*	*Betula pendula*	1	3.3 ± 0.7
T17/29	*T. cerinum*	*Betula pendula*	2	5.3 ± 0.7
T17/30	*T. cerinum*	*Viburnum bodnantense*	2	5.7 ± 0.3
T17/32	*T. atrobrunneum*	*Rhododendron × obtusum* 'amoenum'	0	2.7 ± 0.7
T17/33	*T. hamatum*	*Rhododendron 'moonstone'*	3	6.0 ± 0.0
T17/34	*T. hamatum*	*Rhododendron 'moonstone'*	1	3.3 ± 1.3
T17/35	*T. deliquescens*	*Viburnum bodnantense*	2	5.5 ± 0.4
T17/36	*T. hirsutum*	*Rhododendron × obtusum* 'amoenum'	1	5.7 ± 0.3
T17/37	*T. fertile*	*Viburnum bodnantense*	1	4.7 ± 0.7

T17/38	*T. koningiopsis*	*Rhododendron* × *obtusum* 'amoenum'	2	4.7 ± 0.7
T17/39	*T. cerinum*	*Conifer* sp.	0	4.0 ± 0.0
T17/40	*T. harzianum*	*Conifer* sp.	1	5.0 ± 0.6
T17/41	*T. olivascens*	*Rhododendron* × *obtusum* 'amoenum'	1	4.0 ± 1.2
T17/42 [a]	*T. olivascens*	*Rhododendron* × *obtusum* 'amoenum'	3	6.0 ± 0.0

[a] Indicates the refined selection of *Trichoderma* spp..
