# Supplementary material for: Endophytic Trichoderma spp. can protect strawberry and privet plants from infection by the fungus Armillaria mellea
Source: PLoS One. 2022 Aug 1;17(8):e0271622. doi: 10.1371/journal.pone.0271622 (PMC9342734; doi:10.1371/journal.pone.0271622)
Supplement: S2 Table — (PDF) [file pone.0271622.s003.pdf]

S2 Table. Disease Severity Index (0 – 6 pt. scale) descriptions for *Armillaria mellea* infection of strawberry after three months.

DSI	Above-ground Symptoms	Below-ground Symptoms	Re-isolation
0	No aerial symptoms	No visible *Armillaria* mycelium	Unsuccessful re-isolation of *Armillaria*
1	Aerial symptoms present	No visible *Armillaria* mycelium	Unsuccessful re-isolation of *Armillaria*
2	Aerial symptoms present	No visible *Armillaria* mycelium	Presence of *Armillaria* confirmed by isolation
3	No aerial symptoms	Visible *Armillaria* mycelium colonization	Presence of *Armillaria* confirmed by isolation
4	Aerial symptoms present	Visible *Armillaria* mycelium colonization	Presence of *Armillaria* confirmed by isolation
5	Progressed aerial symptoms	Visible/heavy *Armillaria* mycelium colonization	Presence of *Armillaria* confirmed by isolation
6	Dead plant	Visible/heavy *Armillaria* mycelium colonization	Presence of *Armillaria* confirmed by isolation
