# Supplementary material for: Endophytic Trichoderma spp. can protect strawberry and privet plants from infection by the fungus Armillaria mellea
Source: PLoS One. 2022 Aug 1;17(8):e0271622. doi: 10.1371/journal.pone.0271622 (PMC9342734; doi:10.1371/journal.pone.0271622)
Supplement: S3 Table — Isolation for Armillaria was only made from non-symptomatic tissue, if Armillaria was cultured this was classified as detectable colonization. (PDF) [file pone.0271622.s004.pdf]

S3 Table. Disease Severity Index (0 – 4 pt. scale) descriptions for *Armillaria mellea* infection of privet after nine months. Isolation for *Armillaria* was only made from non-symptomatic tissue, if *Armillaria* was cultured this was classified as detectable colonization.

**DSI**	**Above-ground**	**Below-ground**
0	No aerial symptoms	No visible or detectable *Armillaria* mycelium
1	Aerial symptoms present	No visible or detectable *Armillaria* mycelium
2	No aerial symptoms	Visible or detectable *Armillaria* mycelium colonization
3	Aerial symptoms present	Visible or detectable *Armillaria* mycelium colonization
4	Dead plant	Visible or detectable *Armillaria* mycelium colonization
